# Determination of Zearalenone and Its Derivatives in Feed by Gas Chromatography–Mass Spectrometry with Immunoaffinity Column Cleanup and Isotope Dilution

**DOI:** 10.3390/toxins14110764

**Published:** 2022-11-04

**Authors:** Sunlin Luo, Ying Liu, Qi Guo, Xiong Wang, Ying Tian, Wenjun Yang, Juntao Li, Yiqiang Chen

**Affiliations:** 1 State Key Laboratory of Animal Nutrition, College of Animal Science and Technology, China Agricultural University, Beijing 100193, China; 2 Clover Technology Group Inc., Beijing 100044, China

**Keywords:** zearalenone and its derivatives, GC-MS, immunoaffinity column, isotope dilution, feed

## Abstract

In this study, a gas chromatography–mass spectrometry (GC-MS) method was established for the determination of zearalenone and its five derivatives in feed, including zearalanone, α-zearalanol, β-zearalanol, α-zearalenol, and β-zearalenol. An effective immunoaffinity column was prepared for sample purification, which was followed by the silane derivatization of the eluate after an immunoaffinity chromatography analysis for target compounds by GC-MS. Matrix effects were corrected by an isotope internal standard of zearalenone in this method. The six analytes had a good linear relationship in the range of 2–500 ng/mL, and the correlation coefficients were all greater than 0.99. The limits of detection (LODs) and limits of quantification (LOQs) were less than 1.5 μg/kg and 5.0 μg/kg, respectively. The average spike recoveries for the six feed matrices ranged from 89.6% to 112.3% with relative standard deviations (RSDs) less than 12.6%. Twenty actual feed samples were analyzed using the established method, and at least one target was detected. The established GC-MS method was proven to be reliable and suitable for the determination of zearalenone and its derivatives in feed.

## 1. Introduction

**Figure 1:**
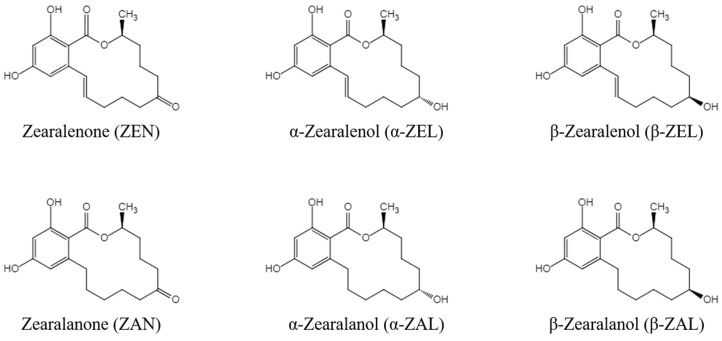
Chemical structures of ZENs.

Zearalenone (ZEN) is a nonsteroidal estrogenic mycotoxin produced by *Fusarium* fungi such as *Fusarium graminearum*, *Fusarium culmorum*, and *Fusarium crookwellense*. It can be metabolized by plants, microorganisms, animals, and humans into many other derivatives and can contaminate animal feed. It is widely found in grains such as corn, wheat, sorghum, and their by-products [1]. The five derivatives of ZEN mainly include zearalanone (ZAN), α-zearalanol (α-ZAL), β-zearalanol (β-ZAL), α-zearalenol (α-ZEL), and β-zearalenol (β-ZEL) [2], and the chemical structures of ZEN and its derivatives (ZENs) are shown in Figure 1. ZENs have estrogen-like effects because of their similar chemical structures to 17 β-estradiol, and α-ZEL has the most pronounced estrogenic activity among the main derivatives [3]. ZENs bind strongly to estrogen receptors and produce estrogen-like effects, leading to disturbances in sex hormone function and reproductive disorders in livestock [4,5]. High doses of ZEN and α-ZEL have adverse effects on the male reproductive system, including significantly reducing sperm counts and production efficiency and serum testosterone concentrations [6]. ZEN and α-ZEL have endocrine-disrupting effects, leading to the disruption of pituitary gonadotropin secretion, which can significantly inhibit the synthesis and secretion of pituitary follicle-stimulating hormone in sows [7]. In addition, high doses of ZEN can induce a series of clinical symptoms in young sows, such as vulvar swelling and vaginal prolapse [7]. Studies in ruminants have shown that even lower intakes of ZEN could affect the ovarian antral follicles of dairy cows and impair their reproductive performance [8]. Other studies confirmed that ZEN also has toxic effects on the offspring of exposed animals. Exposure to ZEN reduced the number of piglets born alive and the litter birth weights of pregnant sows, and it was also reported to affect the reproductive functions of male offspring of pregnant mice via transgenerational cytotoxicity on spermatogonia [9,10]. In addition, ZENs are also hepatotoxic [11,12], genotoxic [13,14], and immunotoxic [15,16]. ZENs can be absorbed by humans or animals through moldy food and feed, and they can seriously harm human health by increasing the risk of human exposure through animal products such as meat, eggs, and milk [17]. Residues of ZENs in agricultural products are a serious human health problem based on their toxic effects. Therefore, it is important to establish an effective detection method for ZENs in feed that would help to produce high-quality feed, improve food safety, and ensure the health of livestock and humans.

To date, many detection technologies have been developed to monitor ZENs in different feed matrices. Immunoassay technologies are reported to be used for rapid detection, such as the enzyme-linked immunosorbent assay and colloidal gold immunochromatography [18,19,20]. Chromatographic techniques are most extensively used for the efficient quantitative analysis of mycotoxins. High-performance liquid chromatography (HPLC) [21,22,23] and liquid chromatography–tandem mass spectrometry (LC-MS/MS) [24,25] have been confirmed to have outstanding performance for the determination of ZENs in feed. Gas chromatography–mass spectrometry (GC-MS) [26,27] and gas chromatography–tandem mass spectrometry (GC-MS/MS) [28] also show excellent analytical performance with low detection limits and great selectivity for ZEN analysis. In addition, some emerging detection technologies have also been applied to the determination of ZENs, including near-infrared detection technology [29], biosensors [30,31], molecular imprinting technology [22,23], etc. Overall, chromatographic techniques, either LC or GC coupled to MS could be the most suitable analytical approaches for the quantitative analysis of ZENs, even at a low concentration level. Compared with the LC-MS method, GC-MS requires derivatization when analyzing polar compounds, and it is time-consuming [28]. Nevertheless, the high machine costs of LC-MS and LC-MS/MS limit their application in many primary laboratories, while GC-MS is often chosen for mycotoxin analysis due to its relatively inexpensive analytical performance.

During mycotoxin analysis, the critical steps are the extraction and purification, and they help to ensure good recoveries of all analytes in a specific feed matrix. Generally, liquid–liquid extraction was performed with organic solvent mixtures of methanol–water or acetonitrile–water, followed by a purification step of solid-phase extraction (SPE) columns or immunoaffinity columns (IAC) [32]. SPE is based on nonspecific binding, and it may cause the coextraction of mycotoxins and other impurities. IAC is an outstanding purification method that relies on the specific binding between an antigen and an antibody [33,34]. Compared with SPE, the immunoaffinity method has the advantages of simple steps, convenience, and a better purification effect, especially for complex feed samples [35,36]. In addition, the derivatization is also a key step for the GC-MS analysis of ZENs. ZENs need be chemically derivatized before analysis by GC-MS because they are all nonvolatile compounds. Some silylation reagents are commonly used for the chemical derivatization of ZENs, including N,O-bis(trimethylsilyl)trifluoroacetamide (BSTFA), N-(trimethylsilyl)imidazole (TSIM), and N-methyl-N-trimethylsilyl trifluoroacetamide (MSTFA), and they were proven to be effective [37].

In addition, a stable isotope dilution would help a lot for mycotoxin analysis in complex matrices such as grain and animal feed. Generally, isotopic internal standards are added to eliminate matrix effects and ensure confidence in analytical results [25]. An isotopically labelled internal standard (ISTD) has physicochemical characteristics very similar to the target analyte, and the changes between the analyte and its ISTD are consistent throughout sample preparation and the machine analysis processes. Therefore, the use of an ISTD could correct the matrix effects during the whole sample analysis and improve the accuracy of mycotoxin quantitative analysis [36,38].

However, there have been few reports about the simultaneous determination of ZENs by the GC-MS method using both immunoaffinity column cleanup and isotope dilution. Hence, the aims of this study were to prepare an IAC specifically for recognizing six ZENs (ZEN, ZAN, α-ZAL, β-ZAL, α-ZEL, and β-ZEL) and to develop a sensitive and accurate GC-MS method based on both immunoaffinity column cleanup and stable isotope dilution for the simultaneous detection of six ZENs in animal feed. This research was expected to provide an effective detection measure for basic laboratories to monitor ZEN contamination in animal feed and ensure feed quality and safety.

## 2. Results and Discussion

### 2.1. Performance of Immunoaffinity Columns (IAC)

#### 2.1.1. Elution Conditions

According to previous reports, the organic solvent methanol was usually used to reduce the polarity of the solution to achieve the purpose of target elution on the IAC [33,39]. Ten milliliters of a phosphate-buffered solution (PBS, 0.1 M, pH 7.0, containing 500 ng of ZEN) was taken and passed through an IAC. After rinsing the IAC with 10 mL of pure water, the elution was performed with 3 mL of 50–100% methanol–water solution, and the eluates under different methanol levels were collected for GC-MS analysis. The elution rate results are shown in Table 1. The elution rate increased gradually with the increase in methanol concentration. When eluted with 90% methanol–water, the elution rate reached 100%. In order to improve the detection sensitivity, 100% methanol was chosen as the eluent for our research.

**Figure 2:**
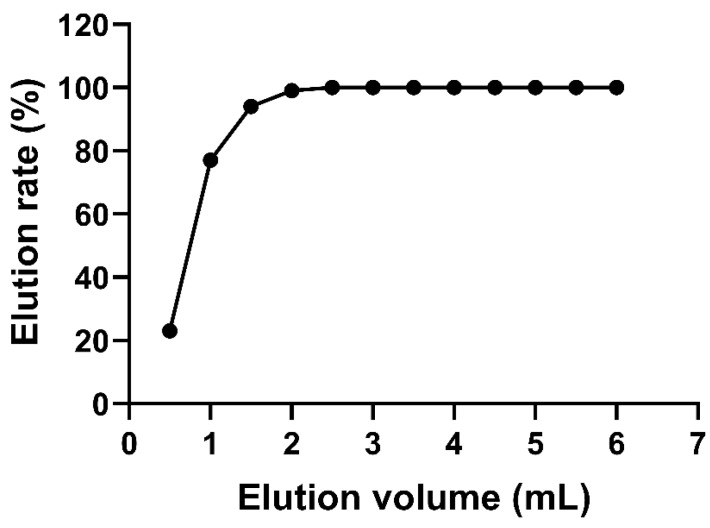
Linear relationship between the mean elution volume and the elution rate.

In addition, the effect of the eluent volume on the elution rate was investigated, and the results are shown in Figure 2. The elution rate increased gradually with the increase in methanol volume. When the methanol volume exceeded 2 mL, the elution rate reached 100%. Hu et al. [40] reported a recovery of 70–110% with an elution condition of 2 mL of methanol. Similarly, 2 mL of methanol was employed to elute the target zeranols in pig muscle, and it achieved recovery values of 74.5–105.0% [41]. In the present study, a 3 mL eluent of methanol was used to achieve sufficient elution effects and obtain good recoveries.

#### 2.1.2. Column Capacity

A ZEN working solution (5 µg of ZEN in 10 mL of PBS) was taken and passed through the IAC. After rinsing with 10 mL of pure water and eluting with 3 mL of methanol, the content of ZEN in the eluate was detected by GC-MS to be 3200 ng, which showed an excellent IAC column capacity. The column capacity of the IAC we prepared was superior to those in the studies by Sun et al. [33] and Hu et al. [40], and it can be used for the purification of sufficient ZEN in actual sample analyses. When samples are contaminated with high levels of ZEN, a dilution step can be performed before the IAC purification.

#### 2.1.3. Specificity

In order to investigate the specificity of the IAC, various types of mycotoxin solutions (200 ng of ZEN, ZAN, α-ZAL, β-ZAL, α-ZEL, β-ZEL, aflatoxin B_1_, ochratoxin A, T-2 toxin, deoxynivalenol, and fumonisins in 10 mL of PBS) were passed through the IAC. After rinsing and eluting, mycotoxin contents were detected. All six ZENs had recoveries above 95%, and several other mycotoxins were not recovered (Table S1). The IAC showed a specific adsorption of ZENs, which indicates that it can be used for the accurate detection of ZENs.

#### 2.1.4. Comparison of Purification Efficiency between SPE and IAC

**Figure 3:**
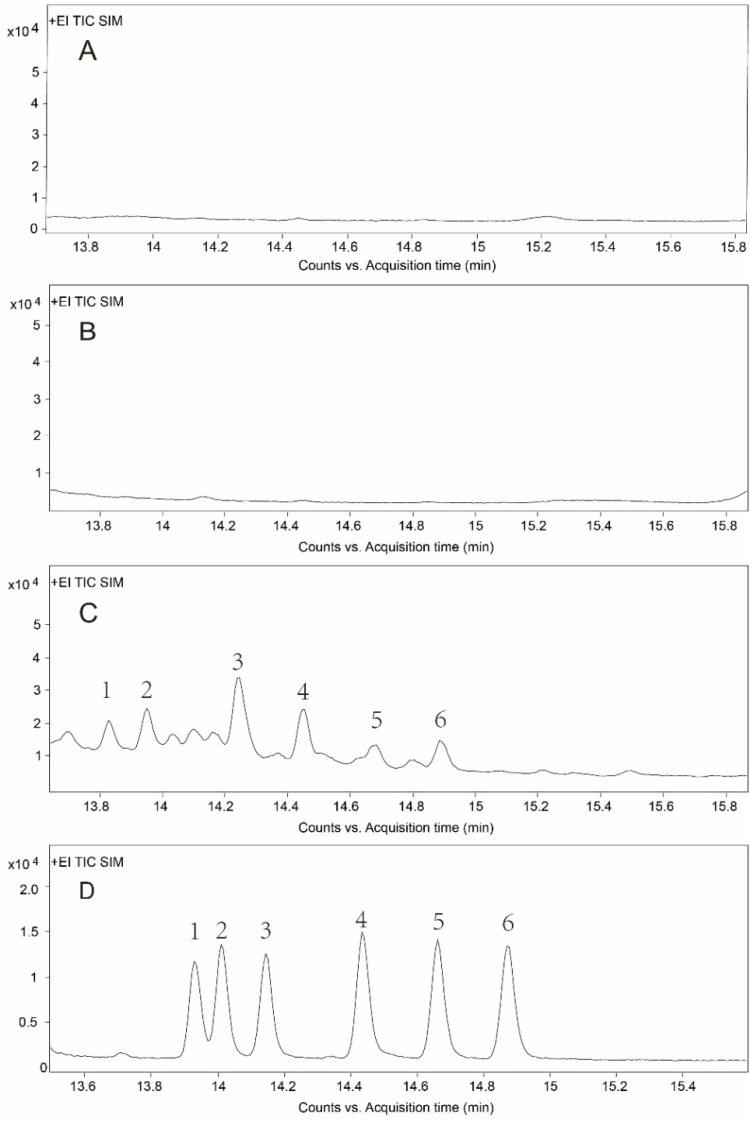
Total ion chromatograms after different cleanups. (**A**) Chromatogram of blank sample after SPE cleanup. (**B**) Chromatogram of blank sample after IAC cleanup. (**C**) Chromatogram of chicken compound feed after SPE cleanup. (**D**) Chromatogram of chicken compound feed after IAC cleanup. The numbers 1–6 in the figure represent ZAN, α-ZAL, β-ZAL, ZEN, α-ZEL, and β-ZEL, respectively.

In order to investigate the differences in purification efficiency of the SPE and IAC cleanup, we compared the mean recovery rates of six compounds in standard solutions (low and high concentrations) and compound feed (pig and chicken) with three replicates (*n* = 3) after SPE and IAC purification. The SPE cleanup operated according to Shen et al. [42]. Table 2 shows that the recoveries ranged from 35.9% to 72.7% for SPE, while IAC cleanup achieved more reliable recoveries ranging from 89.7% to 99.8%. The purification results in our research were similar to a previous report for OTA detection, where the IAC cleanup showed higher and more stable recoveries than the SPE cleanup [43]. In general, IAC cleanup had the advantages of being easier to perform, having fewer purification procedures, and having better specificity and higher purification efficiency in this study compared with SPE. The total ion chromatograms of ZENs in chicken compound feed after different cleanups are shown in Figure 3. The IAC cleanup had better specificity and noticeably lower matrix interference.

#### 2.1.5. Acetonitrile Tolerance

In general, an IAC will tolerate an organic solvent with a low concentration less than 20% [44]. Since this method used acetonitrile–water (80:20, *v*/*v*) to extract samples, in order to ensure that the acetonitrile in the extract did not affect the adsorption of IAC [41] different proportions of the acetonitrile–water mixture (5–40%, 10 mL containing 500 ng of ZEN) were taken and passed through the IAC. After eluting and detecting, the recovery of ZEN was calculated. When the ratio of the acetonitrile–water mixture was lower than 15%, the recovery was higher than 95% (Table S2), which was similar to the results of Hu et al. [40]. To minimize the impact of acetonitrile on IAC, 2 mL of extraction solution was diluted into 30 mL of PBS for purification in this study.

#### 2.1.6. The Adsorption Effect of IAC at Different pH Values

First, 10 mL of PBS with different pH values (pH 3.0, 4.0, 5.0, 6.0, 7.0, 8.0, 9.0, and 10.0, containing 500 ng of ZEN for each) was prepared and passed through the IAC. The optimal adsorption effect was obtained with pH 7.0 PBS, and the recovery of ZEN reached 99.9% under this condition. When the solution was acidic or alkaline, the recovery was lower than 90% (Table S3). Therefore, PBS (pH 7.0) was chosen to dilute the extract for further purification in this research.

#### 2.1.7. Reusage

First, 10 mL of PBS (pH 7.0, containing 500 ng of ZEN) was taken and passed through an IAC, then rinsed, eluted, and detected. The IAC was reused 10 times following this procedure, and the recoveries of ZEN for different times are shown in Table S4. The results showed good recoveries (above 90%) among the 10 usages, indicating that the prepared IAC could be used at least 10 times. The IAC can be reused because the binding of the antigen and antibody is reversible. Antigens and antibodies are bound by noncovalent bonds, with hydrophobic interactions and hydrogen bonds being the two main bonds [43]. Although the immune response is somewhat tolerant to water-soluble organic solvents, when the proportion of organic solvents in the aqueous solution is too high (generally higher than 20%), these bonds are destroyed, resulting in the dissociation of antigens and antibodies [45]. After dissociation, the properties of the antibody remain unchanged, and they can bind to the corresponding antigen again. IACs were used for mycotoxin purification, and they were reported to possess good reusability [46,47]. Hu et al. [40] reused multiple IACs six times to purify mycotoxins in feed samples, and the recovery of target mycotoxins did not decrease. Similarly, the IAC used for the purification of *Fusarium* toxins had a satisfactory recovery after eight cycles of usage [48]. In our study, the prepared IAC showed excellent reusability performance during 10 usages. The more times an IAC can be reused, the lower the cost of analysis. It seems that we have prepared an IAC with excellent performance for the detection of ZENs.

### 2.2. Method Optimization

#### 2.2.1. Optimization of Derivative Conditions

ZENs are nonvolatile compounds that cannot be detected directly after entering a gas chromatograph–mass spectrometer. We were able to obtain volatile derivatives for the analysis by reducing the polarity of the target compounds through silylation derivatization [49]. The previous study reported that the best reaction yields were obtained with N,O-bis(trimethylsilyl)trifluoroacetamide (BSTFA) compared to other silylation agents [37]. In this study, BSTFA (with 1% trimethyl chlorosilane) was used as the derivatization reagent, and the derivatization time and temperature were optimized.

**Figure 4:**
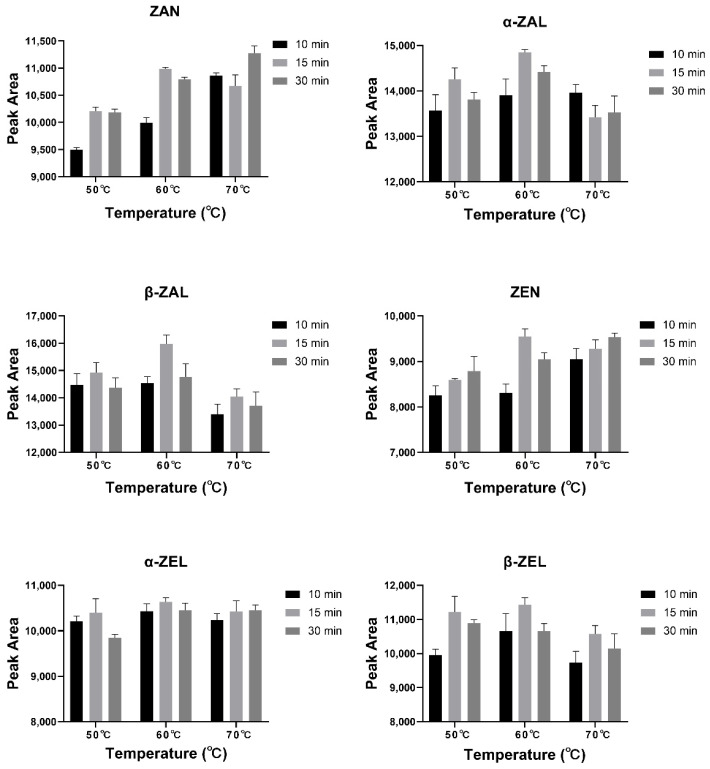
Peak areas of six analytes under different derivatization conditions.

The derivatization times for mycotoxin analysis were generally 10–30 min, and the temperatures were around 60 °C in the previous studies [26,28,42]. Different derivatization temperatures (50, 60, and 70 °C) and derivatization times (10, 15, and 30 min) were set, and a mixed standard solution (100 ng/mL for each mycotoxin) was employed to explore the optimal derivatization conditions for this study (*n* = 3). It was found that five analytes (except ZAN) had the highest peak areas of 9548–15,975 when derivatized at 60 °C for 15 min (Figure 4). ZAN was found to have the highest peak area when derivatized at 70 °C for 30 min, but the average peak areas of 70 °C for 30 min (11,274) and 60 °C for 15 min (10,989) were very close. Hence, a derivatization condition of 60 °C for 15 min was chosen in the present study.

#### 2.2.2. Optimization of Quantitative Ions

**Figure 5:**
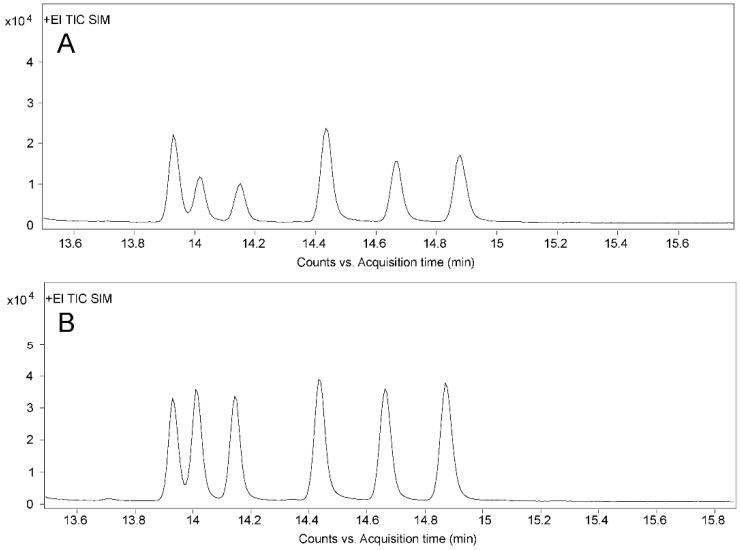
Total ion chromatograms of mixed standard solution. (**A**) Chromatogram before quantitative ion optimization (*m*/*z* 449 for ZAN, *m*/*z* 433 for α/β-ZAL, *m*/*z* 333 for ZEN, and *m*/*z* 305 for α/β-ZEL). (**B**) Chromatogram after quantitative ion optimization (*m*/*z* 307 for ZAN and α/β-ZAL, *m*/*z* 333 for ZEN, and *m*/*z* 305 for α/β-ZEL). The six peaks from left to right are ZAN, α-ZAL, β-ZAL, ZEN, α-ZEL, and β-ZEL, respectively.

The aim of quantitative ion optimization is to select ions with high responses and less interference to improve the detection limit of the method and realize trace analysis. Shen et al. [42] reported that the quantitative ion for ZEN was *m*/*z* 333, while *m*/*z* 433 was chosen in another report [50]. In this study, the optimization of quantitative ions for six analytes was carried on the basis of the mass spectrum parameters from Shen et al. [42]. Through comparing the peak shapes and instrument responsivities of different qualitative ions, *m*/*z* 307 was finally selected as the quantitative ion of ZAN, α-ZAL, and β-ZAL to replace the previous *m*/*z* 449 for ZAN and *m*/*z* 433 for α/β-ZAL. Chromatograms for six analytes performed with different quantification ions are shown in Figure 5. The optimal ionic parameters for all compounds are described in Section 4.4.

#### 2.2.3. Matrix Effect

The chemical properties of ZENs are similar due to their similar chemical structures. Andrade et al. [43] used ^13^C_18_-ZEN ISTD to correct for the matrix effects of ZEN and α-ZEL in the LC-MS/MS analysis. In this study, we attempted to correct for matrix effects to achieve good recoveries using the ^13^C_18_-ZEN ISTD. Moreover, it was more economical to use one ISTD than six ISTDs. Four types of blank feed samples (pig formula feed, pig concentrate feed, beef concentrate supplement, and chicken premix) were spiked with 50 μg/kg of the analytes to assess the use of ^13^C_18_-ZEN ISTD, and absolute recoveries were calculated (Table S5). The results almost showed a matrix-enhancing effect for each feed matrix when ^13^C_18_-ZEN ISTD was not used. After correction with ^13^C_18_-ZEN ISTD, the recoveries were all in the range of 90–112%. Niknejad et al. [28] found that the recovery of ZEN detected by GC-MS/MS was less than 80% without ^13^C_18_-ZEN ISTD. This revealed the necessity of an ISTD when analyzing ZENs, which can effectively correct the matrix effect and significantly improve the quantitative accuracy.

### 2.3. Method Validation

#### 2.3.1. Method Performance

In this study, an IAC-cleanup- and isotope-dilution-based GC-MS method was developed for the determination of ZENs in feed. The performance characteristics were evaluated, including linearity, sensitivity, recovery, and precision, and 20 different feed samples obtained from different feed mills were analyzed for further validation. The linearity was examined by the standard curves of different mixed standard solutions ranging from 2 to 500 ng/mL. The accuracy was examined in terms of recovery (*Rec*) after addition to blank feed samples, as shown in Equation (1). Interday precision was measured over three consecutive days, and intraday precision was tested in quadruplicate for each spiked concentration. The analyte concentration in the actual feed sample (*ω*) was calculated as shown in Equation (2).

*Rec* (%) = *C*_calculated_/*C*_spiked_ × 100 (1)

*C*_calculated_ is the estimated concentration, and *C*_spiked_ is the spiked concentration.

*ω* (μg/kg) = (*ρ*_i_ × *V*_0_ × *V*_2_)/(*m* × *V*_1_) (2)

*ρ*_i_ represents the mass concentration of the analyte in the samples, which is obtained from the standard curve (ng/mL). *V*_0_ represents the volume of the extract (mL). *V*_1_ represents the volume of supernatant pipetted after extraction and centrifugation (mL). *V*_2_ represents the final reconstituted volume after derivatization and dilution (mL). *m* represents the mass of the feed samples (g).

#### 2.3.2. Linearity and Sensitivity

First, 2 µL of ^13^C_18_-ZEN ISTD was added to 1 mL of different mixed standard working solutions with a concentration gradient of 2, 5, 10, 50, 100, 250, and 500 ng/mL for each analyte (the concentration of ^13^C_18_-ZEN was 50 ng/mL in each working solution). The standard solution was analyzed from a low concentration to a high concentration. Standard curves were obtained with the concentration of the compound as the abscissa and the peak area ratio of the compound to the ISTD as the ordinate. The standard curve equations of the ZENs are shown in Table 3. The standard curves of the ZENs showed good linearity, with all linear regression correlation coefficients (R^2^) greater than 0.99.

In order to meet the requirements of trace detection and ensure the quality of feed products, high-sensitivity analytical methods are required. In this study, the limit of detection (LOD) and limit of quantification (LOQ) were monitored based on 3 and 10 times the S/N ratios, respectively. Under the given GC-MS conditions, six targets in different matrices had LODs of 0.40–1.34 μg/kg and LOQs of 1.33–4.46 μg/kg (Table 3). Niknejad et al. [28] reported an LOQ of 8 μg/kg for ZEN GC-MS/MS analysis, and Pack et al. [51] established a GC-MS method for the determination of the ZEN and α-ZEL in swine tissues with an LOQ of 10 ng/g. Compared with those results, the LOQs of the ZENs in our study showed a higher sensitivity, and thus the established GC-MS method could be used for low-level ZEN analysis.

#### 2.3.3. Recovery and Precision

In this study, six noncontaminated matrix samples (chicken formula feed, chicken concentrate feed, chicken premix, pig compound feed, pig premix, and beef concentrate supplement) were spiked with six analytes at low (125 ng/mL), intermediate (250 ng/mL), and high (250 ng/mL) levels for four replicates (*n* = 4). The recoveries were between 89.3% and 112.3% for the ZENs at the three spiked levels, with RSDs ranging from 0.4% to 11.3% (*n* = 4) and from 0.8% to 12.4% (*n* = 3) for intraday and interday precision (Table 4). The recovery and precision results in our study showed excellent accuracy for the quantitative analysis of ZENs. As previously reported, the average recoveries of ZEN were 65–68% in popcorn detected by GC-MS without an ISTD [52], and other mean recoveries of ZEN and α-ZEL were between 75.0% and 120.0% in swine liver and reproductive tissues based on GC-MS [51]. In this study, excellent recoveries were obtained due to the high specificity of the IAC and the matrix effect correction of ^13^C_18_-ZEN ISTD, indicating that the established GC-MS method can be applied to actual feed samples for the analysis of ZENs.

#### 2.3.4. Validation in Naturally Contaminated Feed

To assess the applicability of the established GC-MS method for the detection of six ZENs in different feed samples, 20 feed samples collected from different feed mills in China were analyzed, including 6 pig premix samples, 4 chicken formula feed samples, 5 pig premix samples, and 5 beef concentrate supplement samples. The detection results are shown in Table 5. At least one analyte was detected, and ZEN was found at a 100% detection rate with a maximum concentration of 620.5 μg/kg. In addition, there were occurrences of ZAN, α-ZEL, and β-ZEL in some feed samples, with concentrations ranging from 5.2 to 118.6 μg/kg, suggesting co-contamination or a transformation between ZEN and its derivatives. The relationships between the contents of ZEN and its derivatives in different feed samples require further investigation.

## 3. Conclusions

ZENs are widely found in grains and their by-products, which seriously threatens the growth of animals with estrogen-like effects, and the toxic effects of its derivatives on animals and humans should not be underestimated. Therefore, an accurate and convenient analysis method for ZENs that has wide coverage and high sensitivity is required. This work developed and validated a sensitive and simultaneous GC-MS method based on immunoaffinity column cleanup and isotope dilution for the quantitative determination of ZENs in different feed matrices. The results showed a good linearity, with R^2^ values greater than 0.99 for all ZEN standard curves, and the method performed outstanding recoveries of six analytes in all spiked feed matrices, ranging from 89.6% to 112.3%, suggesting that this method is accurate and reliable for the determination of ZENs in different feed matrices. In addition, 20 feed samples were collected for ZEN analysis. At least one mycotoxin was detected in each sample, and ZAN, α-ZEL, and β-ZEL had certain occurrence. In conclusion, this work provides a reliable detection technology for the rapid and simultaneous determination of ZENs in animal feed. Further studies with larger sample sizes are required to explore the possible relationships between the occurrence and detection contents of ZENs in different feed matrices.

## 4. Material and Methods

### 4.1. Chemicals and Reagents

Standard mycotoxin powders, including ZEN, ZAN, α-ZAL, β-ZAL, α-ZEL, β-ZEL (1 mg dissolved in 1 mL acetonitrile for 1 mg/mL, stored at −20 °C for one year), ^13^C_18_-ZEN ISTD (25.2 µg/mL in acetonitrile, stored at −20 °C for one year), and Sephrose 4B gel (CNBr-activated) were purchased from Sigma-Aldrich (St. Louis, MO, USA). Toluene, HPLC-grade methanol and acetonitrile were purchased from Merck (Darmstadt, Germany). Bis(trimethylsilyl) trifluoroacetamide with 1% Trimethylchlorosilane (BSTFA+1%TMCS) was purchased from Anpel (Shanghai, China) for derivatization. A zearalenone monoclonal antibody was obtained from Clover Technology Group Inc. (Beijing, China). Other analytical-grade chemicals and reagents were obtained from Beijing Chemical Reagent Co. (Beijing, China).

### 4.2. Preparation of Immunoaffinity Columns

Briefly, 1.0 g of CNBr-activated Sepharose 4B was washed with 10 mL of coupling buffer (0.1 M NaHCO_3_, pH 8.3), and the Sepharose 4B was rapidly transferred to an antibody solution (containing 25 mg of ZEN antibody) that had been dialyzed against the coupling buffer for 24 h at 4 °C. Next, the above mixture was fully mixed in an end-over-end manner at room temperature (20–25 °C) for 2 h or at 4 °C overnight. Then, the mixture was centrifuged at 4 °C and 2000 rpm for 1 min, the supernatant was transferred to a new centrifuge tube, and the antibody concentration in the supernatant was measured. Next, the Sepharose 4B at the bottom of the tube was taken and washed with at least five times the volume of matrix-coupling buffer to remove excessive ligand. Then, the matrix was transferred to a 0.1 mol/L Tris-HCl buffer (pH 8.0) for 2 h at room temperature to block all remaining active groups. In order to remove the excessive ligands that were not coupled after coupling, the matrix was washed with a low-pH buffer (0.1 M acetic acid-sodium acetate containing 0.5 M NaCl, pH 4.0) and a high-pH buffer (0.1 M Tris-HCl containing 0.5 M NaCl, pH 8.0) in sequence with at least five times the volume of the matrix for at least three cycles. Finally, the column was wet-packed and stored in 0.01% NaN_3_-PBS.

### 4.3. Sample Preparation

First, 5 g (accurate to 0.0001 g) feed samples were weighed and transferred to a 50 mL polypropylene centrifuge tube, and then 1 g of sodium chloride was added. Subsequently, 20 mL of acetonitrile–water (80:20, *v*/*v*) was added to feed samples and vortexed for 30 min. Then, the samples were centrifuged at 4 °C at 8000 r/min for 10 min, and 2.0 mL of supernatant was diluted with 28.0 mL of PBS (pH 7.0), and 2 µL of ^13^C_18_-ZEN ISTD was added for subsequent purification.

Then, 30 mL of diluted solution was passed through the IAC at a flow rate of 1–2 drops per second until air entered the IAC. Next, 10 mL of water was passed through the IAC until air entered the IAC. In the next step, IAC was rinsed with 3 mL of methanol, and the eluent was collected in a glass test tube. The 3 mL of eluent was dried under nitrogen at 50 °C, and then 200 µL of BSTFA (containing 1%TMCS) was added for derivatization, which was carried at 60 °C for 15 min. When cooled to room temperature, the 200 µL solution was diluted with 800 µL of toluene for GC-MS analysis.

### 4.4. GC-MS Analysis

The GC-MS analysis was carried on an Agilent Technologies 7890A Series gas chromatograph equipped with a 7000A Triple Quad and a 7693 Autosampler (Agilent Technologies, Santa Clara, CA, USA). The DB-5 MS capillary column (0.25 mm × 30 m, 0.25 µm) employed to separate the analytes was purchased from Waters (Milford, MA, USA). The injected sample volume was 2 µL for a single analysis. The injector and detector temperatures were 250 °C and 230 °C, respectively. The oven temperature program was as follows: the initial temperature was 120 °C, and the temperature was increased to 280 °C at a rate of 15 °C/min and held for 5.2 min.

The electron impact ion source (EI) was used, the electron impact energy was 70 ev, the ion source temperature was 230 °C, the interface temperature was 280 °C, the solvent delay was 5 min, and single-ion monitoring mode (SIM) was used with an interval of 0.3 s. The qualitative and quantitative ions of the ZENs are shown in Table 6.

## Figures and Tables

**Table 1:** Effect of the concentration of the methanol solution on the elution rate.

Methanol Concentration in Aqueous Solution (%)	Mean Elution Rate (%)
50	40.1
60	63.6
70	81.2
80	92.5
90	100.0
100	100.0

**Table 2:** Mean recoveries for the six compounds after cleanup by solid-phase extraction (SPE) and immunoaffinity columns (IAC).

Analyte	Matrix	Mean Recoveries	
		SPE (%)	IAC (%)
ZAN	Standard solution	72.7	97.2
	Compound feed	41.7	94.3
α-ZAL	Standard solution	58.5	98.2
	Compound feed	44.9	93.7
β-ZAL	Standard solution	70.4	97.6
	Compound feed	46.7	92.9
ZEN	Standard solution	62.6	99.1
	Compound feed	50.6	91.6
α-ZEL	Standard solution	69.1	96.7
	Compound feed	35.9	89.7
β-ZEL	Standard solution	56.3	98.9
	Compound feed	53.3	91.5

**Table 3:** Parameters of the standard curves, LODs, and LOQs of the ZENs.

Analyte	Linear Range (ng/mL)	Standard Curve	R^2^	LOD (μg/kg)	LOQ (μg/kg)
ZAN	2–500	y = 0.0549x + 0.3195	0.9974	0.50–0.60	1.67–1.99
α-ZAL	2–500	y = 0.0658x + 0.7828	0.9935	0.70–1.21	2.33–4.02
β-ZAL	2–500	y = 0.0747x + 0.7135	0.9937	0.60–1.34	2.00–4.46
ZEN	2–500	y = 0.0562x + 0.3509	0.9982	0.49–0.63	1.63–2.10
α-ZEL	2–500	y = 0.0577x + 0.5182	0.9973	0.40–0.72	1.33–2.39
β-ZEL	2–500	y = 0.0632x + 0.4289	0.9964	0.57–0.90	1.90–3.00

**Table 4:** Recovery and precision of ZENs in feed matrices.

Analyte	Matrix	Mean Recoveries (%) (*n* = 4)	Intraday RSD (%) (*n* = 4)	Interday RSD (%) (*n* = 3)
ZAN	Chicken formula feed	97.3–104.6	2.9–3.4	3.6–7.7
	Chicken concentrate feed	104.2–108.4	2.1–3.0	5.9–6.9
	Chicken premix	102.9–110.3	5.3–9.3	3.6–5.1
	Pig compound feed	94.6–101.0	4.1–7.0	0.8–8.3
	Pig premix	97.1–106.4	0.4–9.5	5.8–10.0
	Beef concentrate supplement	92.1–98.4	3.0–3.6	2.3–9.3
α-ZAL	Chicken formula feed	93.9–104.4	2.5–6.1	4.3–6.8
	Chicken concentrate feed	102.1–106.3	1.6–6.7	5.5–6.9
	Chicken premix	97.2–104.5	1.2–2.7	4.8–9.0
	Pig compound feed	94.3–109.8	2.6–2.9	3.2–11.7
	Pig premix	98.6–103.4	3.3–3.4	2.1–4.6
	Beef concentrate supplement	92.6–105.2	3.9–6.1	5.2–12.4
β-ZAL	Chicken formula feed	93.6–98.8	1.5–2.0	7.1–9.7
	Chicken concentrate feed	98.3–105.7	2.2–2.3	3.4–4.2
	Chicken premix	97.5–100.3	0.8–2.9	1.5–6.9
	Pig compound feed	101.2–107.1	4.1–6.1	5.1–7.3
	Pig premix	97.3–100.3	1.9–3.5	1.9–8.6
	Beef concentrate supplement	92.4–103.7	0.6–7.5	5.2–10.0
ZEN	Chicken formula feed	97.8–103.6	1.4–5.4	3.8–7.6
	Chicken concentrate feed	98.6–103.7	4.8–4.9	2.9–7.9
	Chicken premix	101.2–104.6	1.3–5.4	2.6–5.7
	Pig compound feed	102.6–110.4	4.8–10.7	0.8–8.8
	Pig premix	94.3–98.9	3.1–10.0	5.2–10.2
	Beef concentrate supplement	96.2–103.2	2.4–3.1	2.7–9.0
α-ZEL	Chicken formula feed	91.5–101.1	0.6–4.9	6.9–11.8
	Chicken concentrate feed	98.6–101.3	0.4–4.6	4.7–9.1
	Chicken premix	91.4–97.6	0.3–5.9	2.9–7.1
	Pig compound feed	93.6–110.2	1.6–9.7	2.1–9.2
	Pig premix	89.6–94.9	3.4–5.2	6.5–10.5
	Beef concentrate supplement	91.3–102.6	1.8–3.6	5.7–5.8
β-ZEL	Chicken formula feed	96.3–112.3	1.4–7.7	7.6–9.8
	Chicken concentrate feed	97.8–99.6	1.9–3.7	4.4–8.6
	Chicken premix	93.6–99.3	1.9–5.2	3.2–11.5
	Pig compound feed	94.0–105.2	3.8–6.2	8.9–10.4
	Pig premix	95.1–98.3	2.0–6.1	2.5–6.0
	Beef concentrate supplement	90.4–104.1	0.5–11.3	6.3–10.3

**Table 5:** Determination results of ZENs in feed samples.

Analyte	Detection Rate ^1^ (%)	Minimum ^2^/Maximum (μg/kg)
ZAN	30	5.2/8.9
α-ZAL	0	-
β-ZAL	0	-
ZEN	100	19.8/620.5
α-ZEL	45	9.1/111.4
β-ZEL	50	5.8/118.6

^1^ Proportion of samples with concentration > LOQ. ^2^ Minimum detectable concentration of detectable samples.

**Table 6:** Qualitative and quantitative ions of ZENs.

Analyte	Retention Time (min)	Qualitative Ion (*m*/*z*)	Quantitative Ion (*m*/*z*)
ZAN	13.943	449, 450, 335, 307	307
α-ZAL	14.024	523, 433, 335, 307	307
β-ZAL	14.158	523, 433, 335, 307	307
ZEN	14.441	462, 429, 333, 305	333
α-ZEL	14.677	536, 431, 333, 305	305
β-ZEL	14.885	536, 431, 333, 305	305
^13^C_18_-ZEN	14.441	344, 316, 447, 480	344

## Data Availability

Not applicable.

## Supplementary Materials

The following supporting information can be downloaded at: https://www.mdpi.com/article/10.3390/toxins14110764/s1, Table S1: Recoveries of mycotoxins from the spiked water; Table S2: Acetonitrile tolerance of IAC; Table S3: Recoveries of IAC at different pH values; Table S4: Recovery after reuse of IAC; Table S5: Recovery of six compounds in different feeds with and without isotopic dilution.
