# Supplementary material for: Determination of Zearalenone and Its Derivatives in Feed by Gas Chromatography–Mass Spectrometry with Immunoaffinity Column Cleanup and Isotope Dilution
Source: Toxins (Basel). 2022 Nov 4;14(11):764. doi: 10.3390/toxins14110764 (PMC9697342; doi:10.3390/toxins14110764)
Supplement: Supplementary file 1 [file toxins-14-00764-s001.zip › toxins-1937534-Supplementary.pdf]

Determination of Zearalenone and Its Derivatives in Feed by Gas Chromatography-Mass Spectrometry with Immunoaffinity Column Cleanup and Isotope Dilution

Sunlin Luo[1†], Ying Liu [1†], Qi Guo[2], Xiong Wang[2], Ying Tian[1], Wenjun Yang[1], Juntao Li[1,*] and Yiqiang Chen[1,*]

1 State Key Laboratory of Animal Nutrition, College of Animal Science and Technology , China Agricultural University , Beijing 100193, China

2 Clover Technology Group Inc, Beijing 100044, China

* Corresponding author: Juntao Li and Yiqiang Chen, Email: lijuntao@cau.edu.cn (J.L.); yqchen@cau.edu.cn (Y.C.)

† Both authors contributed equally to the manuscript

Table S1. Recoveries of mycotoxins from the spiked water.

Mycotoxins	Recovery (%)
ZAN	96.8
α-ZAL	95.9
β-ZAL	97.6
ZEN	100.0
α-ZEL	98.7
β-ZEL	98.9
Aflatoxins B_1	0
Ochratoxin	0
T-2 toxin	0
Deoxynivalenol	0
Fumonisins	0

Table S2. Acetonitrile-tolerance of IAC.

Acetonitrile concentration in aqueous solution (%)	5%	10%	15%	20%	25%	30%	35%	40%
Recovery **(%)**	99.6	98.5	96.7	94.6	92.1	79.6	68.0	46.2

Table S3. Recovery of IAC at different pH values.

pH	3.0	4.0	5.0	6.0	7.0	8.0	9.0	10.0
Recovery (%)	13.5	36.5	67.6	86.7	99.9	85.1	55.3	36.8

Table S4. Recovery of Reuse IAC.

Times	1	2	3	4	5	6	7	8	9	10
Recovery (%)	100	99.2	97.1	98.2	96.6	95.2	93.2	92.1	92.8	92.1

Table S5. Recovery of six analytes in different feed matrices with and without isotope dilution.

Analyte	Matrix	Recovery with isotope dilution (%)	Recovery without isotope dilution (%)
ZAN	Pig formula feed	123.25	105.6
	Pig concentrate feed	135.72	109.8
	Beef concentrate supplement	114.73	102.0
	Chicken premix	110.06	97.1
α-ZAL	Pig formula feed	119.90	103.1
	Pig concentrate feed	122.12	108.9
	Beef concentrate supplement	122.98	109.6
	Chicken premix	117.52	100.8
β-ZAL	Pig formula feed	131.88	109.7
	Pig concentrate feed	134.18	110.1
	Beef concentrate supplement	123.79	104.6
	Chicken premix	121.47	101.1
ZEN	Pig formula feed	138.42	110.5
	Pig concentrate feed	128.10	104.3
	Beef concentrate supplement	115.72	102.2
	Chicken premix	129.38	103.7
α-ZEL	Pig formula feed	123.72	96.5
	Pig concentrate feed	130.22	103.6
	Beef concentrate supplement	119.08	95.0
	Chicken premix	117.77	93.6
β-ZEL	Pig formula feed	123.54	102.2
	Pig concentrate feed	135.01	107.5
	Beef concentrate supplement	121.92	101.3
	Chicken premix	121.03	99.1
